# Clinical Implementation of Robust Optimization for Craniospinal Irradiation

**DOI:** 10.3390/cancers10010007

**Published:** 2018-01-03

**Authors:** Alexandria Tasson, Nadia N. Laack, Chris Beltran

**Affiliations:** Department of Radiation Oncology, Mayo Clinic, Rochester, MN 55905, USA; tasson.ali@mayo.edu (A.T.); laack.nadia@mayo.edu (N.N.L.)

**Keywords:** craniospinal irradiation, CSI, robust optimization, proton spot scanning

## Abstract

With robust optimization for spot scanning proton therapy now commercially available, the ability exists to account for setup, range, and interfield uncertainties during optimization. Robust optimization is particularly beneficial for craniospinal irradiation (CSI) where the large target volume lends itself to larger setup uncertainties and the need for robust match lines can all be handled with the uncertainty parameters found inside the optimizer. Suggested robust optimization settings, parameters, and image guidance for CSI patients using proton therapy spot scanning are provided. Useful structures are defined and described. Suggestions are given for perturbations to be entered into the optimizer in order to achieve a plan that provides robust target volume coverage and critical structure sparing as well as a robust match line. Interfield offset effects, a concern when using multifield optimization, can also be addressed within the robust optimizer. A robust optimizer can successfully be employed to produce robust match lines, target volume coverage, and critical structure sparing under specified uncertainties. The robust optimizer can also be used to reduce effects arising from interfield uncertainties. Using robust optimization, a plan robust against setup, range, and interfield uncertainties for craniospinal treatments can be created. Utilizing robust optimization allows one to ensure critical structures are spared and target volumes are covered under the desired uncertainty parameters.

## 1. Introduction

Craniospinal irradiation (CSI), particularly for pediatric patients, is one of the most important treatment sites for proton therapy [1,2,3,4,5,6]. These patients have large treatment volumes that require extensive treatment planning time to create a robust treatment plan to ensure that the target volume is covered under setup uncertainties. Now that robust optimization for spot scanning proton therapy is available, robustness can be accounted for during the optimization process rather than it being a retrospective analysis after the dose calculation. 

Creating a dose gradient that spans approximately 10 cm between two matched fields results in a match line that is robust to interfield uncertainties. Prior to robust optimization, the only way to achieve this dose gradient was to create numerous optimization structures that forced the dose to step down incrementally within the match [7]. Each field needed to be optimized individually and then summed together. With robust optimization, all of the fields can be optimized together and the optimizer will automatically form this dose gradient without the need for additional structures.

## 2. Results

A robust optimizer can successfully be employed to produce robust match lines, target volume coverage, and critical structure sparing under specified uncertainties for CSI treatments. The robust optimizer can also be used to reduce the effects arising from interfield uncertainties. Suggested settings to be used in the optimizer are listed in Table 1.

## 3. Discussion

A robust optimizer provides the ability to actively account for setup, range, and interfield uncertainties for CSI during the optimization. Prior to having this capability, robustness of a plan was evaluated after the final dose calculation, and if the robustness of a plan was not satisfactory, it meant returning to the optimizer to make adjustments to the plan. Being able to robustly optimize not only saves time, but also reduces frustration for the treatment planner.

Prior to robust optimization, in order to create a large gradient in the matching region, it was necessary to use many structures in order to communicate to the optimizer what the desired dose distribution was. Each isocenter needed to be optimized independently and then summed with the hope that there would not be any hotspots. Now all one needs to do is enter the appropriate field perturbation, and all of the fields are optimized at the same time, which also increases efficiency.

One of the benefits of having a robust match line is that it allows for some additional flexibility when lining up the patient, namely each isocenter can be matched independently. This saves time in the treatment room, since it is no longer necessary to line up the entire treatment volume prior to treating the first field. 

A robust optimizer also provides the necessary tools to mitigate interfield effects. This leads to a more homogeneous dose distribution and reduces the potential for undesired hotspots when evaluating robustness. We have successfully planned and treated over 60 craniospinal patients using the large dose gradient matching technique, and more than half of those patients were planned using robust optimization.

## 4. Materials and Methods 

### 4.1. Creating Structures

A number of structures needed for robust optimization will be referenced throughout this paper and will first be defined. The tools used are typically standard in most treatment planning systems, in this description the Eclipse version 13.7 (Varian Medical Systems, Palo Alto, CA, USA) was used. The optimization target volume (OTV) is the primary structure that will be used for optimization. The OTV is typically equal to the clinical target volume (CTV) but may include an expansion. The scanning target volume (STV) is the structure used to define where spots can be placed, and is specified for each field independently. The planning organ at risk volume (PRV) is an expansion on a critical structure used to control dose to a critical structure. A planning target volume (PTV) is not used [8] since the CTV coverage is evaluated directly under various uncertainties. These are the general meaning of these structures. Below, we outline the specific margins used to construct these structures for CSI treatments.

The beam arrangement used for CSI treatments is typically two fields treating the brain. An anterior superior field and a posterior field are preferred since this arrangement often results in better coverage of the cribriform with lower doses to the eyes and cochlea, as well as less biologic enhancement to the optic structures since this field does not range out in these structures. It may be advantageous if the anterior superior field does not treat the entire brain, since this could lead to minimum monitor issue (MU) issues. For the spine, a single posterior field per isocenter is used. Shifts between isocenters are set such that the left–right and anterior–posterior coordinates are unchanged between isocenters.

Due to the large size of the treatment field, setting up and aligning CSI patients is often a lengthy and tedious task. Because of this, larger setup uncertainties are used in the spine fields. Setup uncertainties of 3 mm are planned for in the brain and 5 to 7 mm of setup uncertainties are planned for in the spine. The OTV used for a craniospinal patient is equal to the CTV in the brain region and for the spine region, an additional 2 to 4 mm are added in the left/right and ant/post direction, see Table 1. These extra margins in the spine fields are required due to the complexity of aligning the entire spinal column as many patients have notable curvature, potential growth, and compression or decompression of the spinal column. Since a different setup uncertainty is desirable in the spine than in the brain, the use of an expanded OTV in the spine allows for one set of ‘plan perturbations’ to be used in robust optimization. 

**Figure 1:**
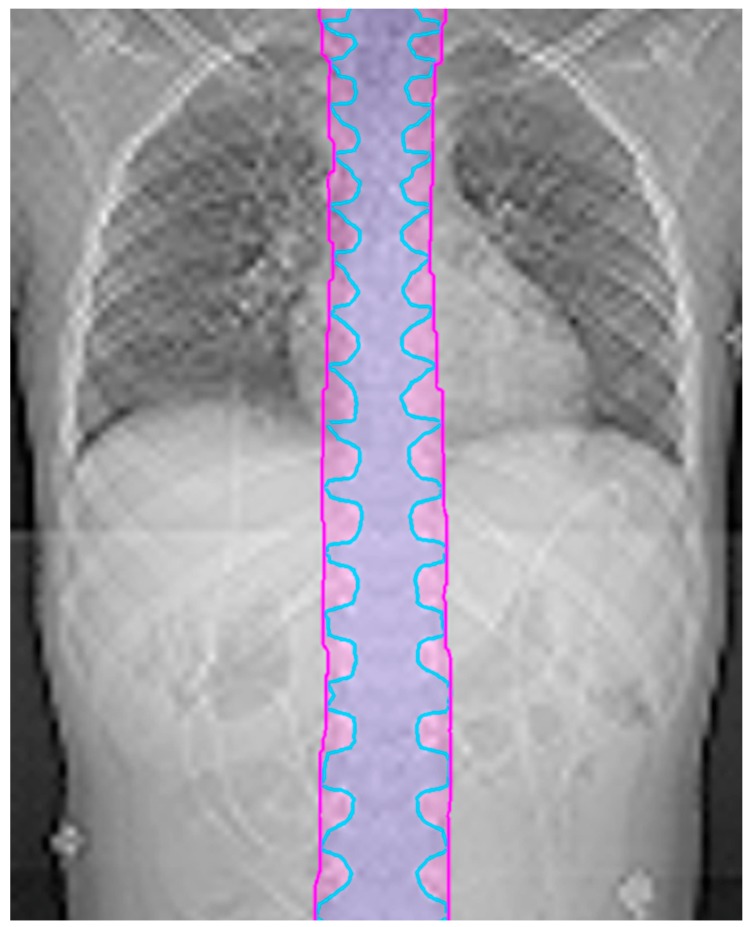
The Clinical Target Volume (CTV) (blue) is scalloped. The Optimization Target Volume (OTV) (magenta) is equal to the CTV with a 10 mm expansion in the superior/inferior direction.

Since the CTV in the spine typically follows the nerve roots, the CTV and thus OTV can look scalloped in the coronal plane. The optimizer may try to chase this contour, which frequently leads to undesired hot spots. To mitigate this issue, the margin for the OTV in the spine can be extended in the superior/inferior directions by 10 mm to create a more continuous target volume. An example is shown in Figure 1. This final OTV structure, including the spine expansion and de-scalloping, is referred to as the OTV all.

A field specific OTV (fs_OTV) is equal to the overall OTV but only in the area that will be treated by the designated field(s). Thus, an fs_OTV is created for each isocenter or field if not all of the fields for a given isocenter are treating the same volume. The fs_OTVs overlap adjacent fs_OTVs in the spine by at least 100 mm to allow for the development of a robust matching region.

These fs_OTVs are the base structures used to create the STVs. For robust optimization, the recommended expansion for an STV from the fs_OTV perpendicular to the beam direction is the positional uncertainty value used during optimization plus the spot spacing. The spot spacing is generally 1.0 to 1.5 times the spot size (1 sigma) in the patient. In our clinic, the positional uncertainty is 3 mm and the spot spacing is 5 mm. Thus, the STV for each field is the fs_OTV plus an 8 mm expansion perpendicular to the beam direction, the STV expansion in the beam direction is equal to the range uncertainty, which is typically 3% to 4%.

There are additional structures that will aid in the optimization process. The small section of brain near the cribriform plate is in an inhomogeneous area that requires good dose coverage. By contouring the ‘cribriform brain’, it is easier to achieve and evaluate the dose coverage and robustness of this area. In young patients, this ‘cribriform brain’ section extends anteriorly and is subject to under-coverage. 

**Figure 2:**
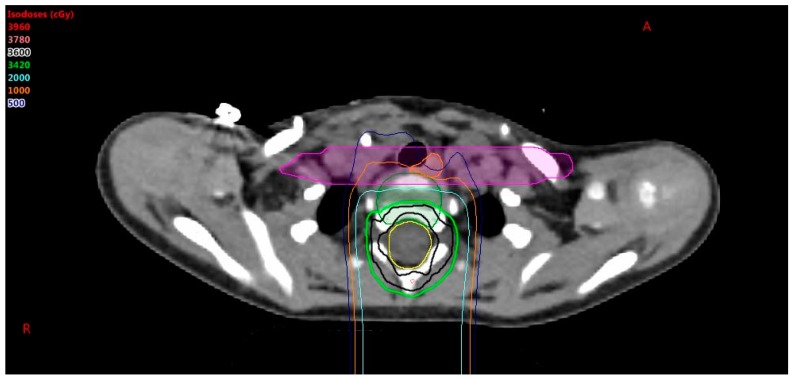
When compromising vertebral body coverage to spare the esophagus (orange circle), it is desired to have the dose form a straight line across the vertebral body. A box drawn around the esophagus (purple shaded line/area) is used to create this dose distribution. Note the prescription is 36 Gy.

In younger patients, where it is recommended that the entire vertebral body be treated to promote uniform growth [9], it is possible that nearby critical structures will get an undesired dose. A higher priority is given to esophagus and constrictor sparing than vertebral body coverage. When vertebral body coverage is forfeited in lieu of critical structure sparing, it is desired to have the dose form a straight line across the vertebral body, as shown in Figure 2, to promote uniform lateral growth of the bone. The pros and cons of sparing the esophagus vs. symmetric anterior to posterior irradiation of the vertebral body must be considered. To achieve this, a non-uniform PRV is created by forming a box around the esophagus and constrictors with expansions of 5 mm anterior–posterior and 5 cm left–right. The dose is pushed out of this PRV box structure to achieve the lowest dose possible to the esophagus and constrictors without affecting CTV coverage, noting that the vertebral body will have an anterior–posterior gradient, but not a right–left gradient. The esophagus sparing is important for high risk patients with a prescription of 36 GyE, but may be less so for low risk patients with a prescription less than 24 GyE.

### 4.2. Robust Optimization Parameters

There are two types of perturbation parameters that can be entered into robust optimization systems: systematic plan perturbations and field specific perturbations. The plan perturbations result in the optimizer shifting all isocenters by the designated amount. Range uncertainties are also entered as plan perturbations, since it is a parameter that is applied to the entire plan and not an individual field. A field perturbation is a shift of the designated field(s), primarily accounting for interfield motion of the patient.

The desired range uncertainty is entered as a plan perturbation, typically 3%. Positive and negative plan perturbations of 3 mm are entered for each coordinate axis independently. It is not necessary to robustly optimize the vertebral body coverage. 

**Figure 3:**
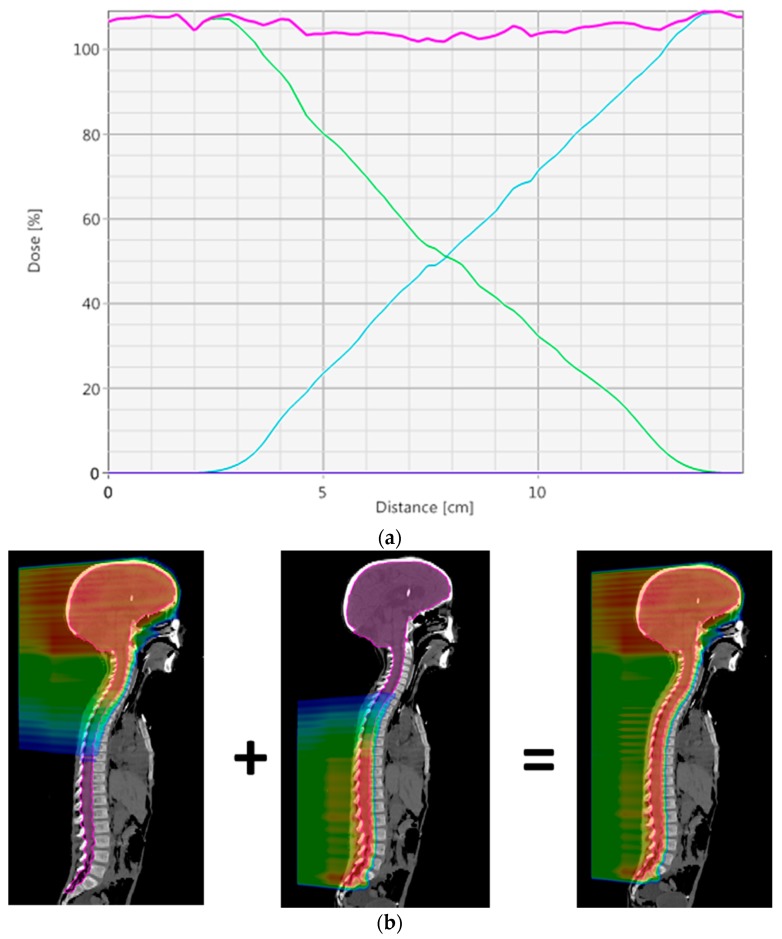
(**a**) Dose profile through a matching area. The green and blue lines are the doses for individual fields. The magenta line is the sum of the two fields; (**b**) dose color wash showing the dose gradients for the superior field (left) and inferior field (middle), and sum (right).

To create a robust match line, a dose gradient where the dose is uniformly reduced from 100% to 0% over approximately 100 mm is desired. This gradient will result in a 1% change in dose for every 1 mm offset between fields, meeting a general requirement for CSI that a 5 mm interfield shift will not lead to more than a 5% hot or cold spot. This matching technique is similar to what is described by Lin et al. [7], except there a manual technique is used to get the desired gradient. To achieve this in the robust optimization setting, an inter-field shift of 5 mm in the superior–inferior direction for one of each field in the match region is required; see field perturbation in Table 1. Figure 3 shows an example of the dose profile through a match and the dose color wash for two treatment fields that are used to create the match. By robustly optimizing, this optimal dose distribution in the match will be automatically created.

### 4.3. Optimization Parameters

The last thing to be determined before starting the optimization is which structures will be robustly optimized. The OTV_all will be robustly optimized, as well as the “cribriform brain”. Even though the “cribriform brain” is included in the OTV, it is a small structure in an area that is difficult to dosimetrically cover, and because the OTV_all is much large, it is likely that degradation in dose in this area will not be detected by looking at the OTV_all dose volume histogram. 

It is important to remember that robust optimization is not only used to ensure coverage of a target structure, but it can also be used to ensure that hotspots remain outside of critical structures under various uncertainties. This is particularly important for optic structures and brainstem, since they are frequently adjacent to boost volumes for CSI patients. The cochlea is another structure where robust optimization may be desired though this may result in loss of coverage to the surrounding CTV. Note that if robust optimization is used on a PRV, this will result in an additional margin being added to the PRV, which is usually not necessary.

Sparing critical structures in a robust way can be done using two methods. Both methods will successfully push the dose away from the critical structure, ensuring that it will be protected despite setup uncertainties. The first approach is to use PRVs. Typical expansions are 2 to 5 mm from the critical structure. The other option is to use the robust optimizer to create a similar dose buffer around the critical structure. There are a couple of benefits to using the robust optimizer. First, PRVs will not be needed, which will save contouring time. Another benefit is that the optimizer will determine the necessary boundaries needed to protect the structure. The margins created by the robust optimizer are often smaller than what would have been used with a PRV. This approach will also allow range uncertainties to be explicitly accounted for in the optimization.

Once the plan has finished calculating, in addition to the typical items that are looked at (i.e., nominal target volume coverage and critical structure doses) there are a number of additional questions to be considered when reviewing the plan.

1. When evaluating the plan uncertainty dose volume histograms (DVH), is there sufficient coverage of and acceptable hotspots in the CTV?
  - A typical value used to judge robustness is if each plan uncertainty DVH has greater than 95% of the dose covering 95% of the target.
  - Ensure that small regions, such as the cribriform, maintain good coverage under plan uncertainties.
2. When evaluating the plan uncertainty DVHs, have critical structures been spared adequately and are potential hotspots acceptable?
  - Hot spots of 5–7% above the nominal plan are to be expected for serial type structures.
3. Does the dose profile through the matching area(s) show a steady decrease/increase of dose from the contributing fields that spans the whole desired matching area?

### 4.4. Image Guidance 

Given a robust match line, one can image each isocenter independently, thereby improving efficiency. For the brain fields, the cribriform plate, base of skull, and the most inferior vertebral body (usually lower c-spine) are matched with a 2 mm tolerance. Care must be taken with the tilt of the head to ensure proper coverage of the target and sparing of critical structures. 

After the superior (brain) fields are treated, the patient is shifted a predetermined amount. The vertebral body that was used in the brain fields for image guidance is used once again, along with a vertebral body near the inferior border of this new spine field, with a tolerance of 3 mm. 

After that field is treated, the patient is shifted inferior once again to the next isocenter and the process is repeated until all fields are treated. It is not uncommon to have one brain isocenter and up to three spine isocenters for very tall patients. 

## 5. Conclusions

A robust optimizer provides the necessary tools to mitigate interfield effects, which leads to a more homogeneous dose distribution and reduces the potential for undesired hotspots when evaluating plan robustness. With the presented method, planning for CSI treatments is efficient and robust.

## Figures and Tables

**Table 1:** A summary of recommended settings for robust optimization of craniospinal irradiation.

Action	Recommendation
OTV Expansion from CTV	0 mm (OTV = CTV) in the brain region; 10 mm sup/inf in the spine to eliminate scalloping; 2 to 4 mm right/left and ant/post in the spine region.
STV Expansion from fs_OTV	Desired robustness + spot spacing (i.e., 3 mm + 5 mm), 3% range
Plan Perturbations	+/− 3%, +/− 3 mm in each direction
Field Perturbations	5 mm sup/inf for each field in the match regions.
Robustly optimized structures	OTV, cribriform brain, optic structures, brainstem, cochlea (optional)

fs OTV: field specific Optimization Target Volume; CTV: Clinical Target Volume; STV: Scanning Target Volume. +/−: plus/minus.

## References

[1] Brodin N.P., Rosenschöld P.M.A., Aznar M.C., Kiil-Berthelsen A., Vogelius I.R., Nilsson P., Lannering B., Björk-Eriksson T. (2011). Radiobiological risk estimates of adverse events and secondary cancer for proton and photon radiation therapy of pediatric medulloblastoma. Acta Oncol..

[2] Yoon M., Shin D.H., Kim J., Kim J.W., Kim D.W., Park S.Y., Lee S.B., Kim J.Y., Park H.J., Park B. (2011). Craniospinal irradiation techniques: A dosimetric comparison of proton beams with standard and advanced photon radiotherapy. Int. J. Radiat. Oncol. Biol. Phys..

[3] Zhang R., Howell R.M., Giebeler A., Taddei P.J., Mahajan A., Newhauser W.D. (2013). Comparison of risk of radiogenic second cancer following photon and proton craniospinal irradiation for a pediatric medulloblastoma patient. Phys. Med. Biol..

[4] Brown A.P., Barney C.L., Grosshans D.R., McAleer M.F., De Groot J.F., Puduvalli V.K., Tucker S.L., Crawford C.N., Khan M., Khatua S. (2013). Proton beam craniospinal irradiation reduces acute toxicity for adults with medulloblastoma. Int. J. Radiat. Oncol. Biol. Phys..

[5] Clair W.S., Adams J.A., Bues M., Fullerton B.C., La Shell S., Kooy H.M., Loeffler J.S., Tarbell N.J. (2004). Advantage of protons compared to conventional X-ray or IMRT in the treatment of a pediatric patient with medulloblastoma. Int. J. Radiat. Oncol. Biol. Phys..

[6] Yuh G.E., Loredo L.N., Yonemoto L.T., Bush D.A., Shahnazi K., Preston W., Slater J.M., Slater J.D. (2004). Reducing toxicity from craniospinal irradiation: Using proton beams to treat medulloblastoma in young children. Cancer J..

[7] Lin H., Ding X., Kirk M., Liu H., Zhai H., Hill-Kayser C.E., Lustig R.A., Tochner Z., Both S., McDonough J. (2014). Supine craniospinal irradiation using a proton pencil beam scanning technique without match line changes for field junctions. Int. J. Radiat. Oncol. Biol. Phys..

[8] ASTRO Model Policies: Proton Beam Therapy (PBT). http://www.astro.org/uploadedFiles/Main_Site/Practice_Management.

[9] McMullen K., Buchsbaum J., Douglas J., McDonald M., Johnstone P. (2013). Growth abnormalities of the spine after radiation therapy: Respecting the past while moving forward in proton craniospinal irradiation. Pract. Radiat. Oncol..

